# Short and Efficient Synthesis of Alkyl- and Aryl-Ortho-Hydroxy-Anilides and their Antibiotic Activity

**DOI:** 10.3797/scipharm.1401-19

**Published:** 2014-03-13

**Authors:** Jürgen Krauß, Eva Plesch, Sabine Clausen, Franz Bracher

**Affiliations:** Department of Pharmacy – Center for Drug Research, Ludwig-Maximilian-University, Butenandtstr. 5–13, 81377 Munich, Germany.

**Keywords:** Anilides, Hydrogenation, Antibiotic activity, Aminolysis

## Abstract

Ortho-hydroxy-anilides are part of natural products like the new antibiotics platencin (A) and platensimycin (B). An important step in the total synthesis of these antibiotics or their derivatives is the preparation of the *o*-hydroxy-anilide partial structure. The presented method allows the preparation of *o*-hydroxy-anilides and *o*-dihydroxy-anilides from 2-nitrophenol esters in a one-step synthesis without protecting the hydroxy group. Aryl- and alkyl-anilides were prepared following this method as simple analogues of platensimycin (A). The resulting compounds were tested in an agar diffusion assay for their antibiotic potency.

## Introduction

**Fig. 1.**
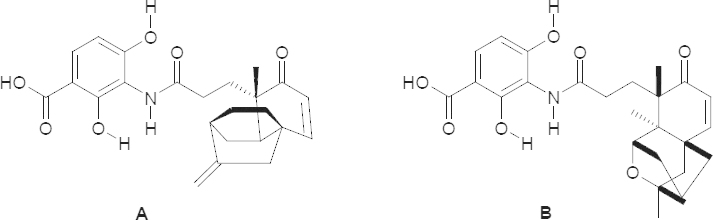
Structure of platencin (A) and platensimycin (B)

The 3-amino-2,4-dihydroxybenzoic acid core is an essential part of the new antibiotic drugs platencin (**A**) and platensimycin (**B**) [Figure 1], which show a high activity against Gram-positive bacteria, especially methicillin-resistant *Staphylococcus aureus* (MRSA) or vancomycin-resistant enterococci (VRE). Platencin (**A**) shows MIC values against MRSA of about 0.1 μg/mL and platensimycin (**B**) 0.2–0.4 μg/mL against MRSA and 0.4–0.8 μg/mL against VRE. Furthermore, platensimycin (**B**) shows low toxicity against mammalian cells (IC_50_ > 1000 μg/mL in HeLa cells).

**Fig. 2.**
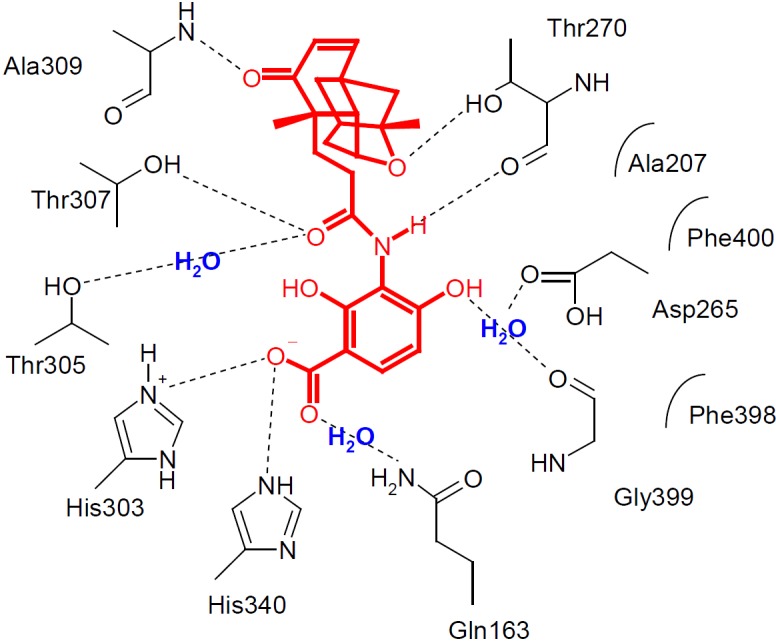
Schematic diagram showing the key interactions between platensimycin and the FabF enzyme [4]

Platencin and platensimycin show a new mechanism of action by inhibiting the bacterial fatty acid synthesis. Bacterial fatty acid synthesis is carried out by fatty acid synthase (FAS II). Each step in this synthesis is encoded by separate proteins. A key step in the pathway is the condensation of the acyl-enzyme intermediate and malonyl-acyl carrier protein by catalysis of the FabF–enzyme towards the ß-ketoacyl-acyl carrier protein (elongation of the fatty acid chain). Both natural products inhibit the FabF-enzyme (Fig. 2) [1–4].

The total synthesis of both natural products is long and expensive. In the last few years, a lot of total syntheses of platencin (**A**) and platensimycin (**B**) were published. Even the synthesis of the 3-amino-2,4-dihydroxy benzoic acid partial structure, which is essential for binding to the enzyme FabF (Figure 2), takes several steps in these total syntheses [5–13]. In continuation of our work on simple platensimycin analogues [14, 15], we hereby present the short and effective preparation of the *o*-hydroxy-anilide partial structure without the requirement of a protecting group.

## Results and Discussion

In the first series, commercially available 4-hydroxy-3-nitrobenzoic acid (**1**) was esterified with methanol / H_2_SO_4_ following a standard protocol to give the methyl ester **2** [14]. The phenol group of the methyl ester **2** was esterified with several aromatic or aliphatic carboxylic acid chlorides to give the phenol esters **3a**–**h**. The following hydrogenation of the nitro group with Pd/C (5%) in methanol led to the amino group which reacted in the same procedure under aminolysis of the phenol ester to the resulting anilides **4a**–**g** [14–18]. The olefin partial structures of **3a** and **3f** were hydrogenated under these conditions but the halogen substituent of **3b** was stable. Reaction of **3h** led only to a mixture of the products that couldn´t be separated by flash column chromatography.

**Sch. 1.**
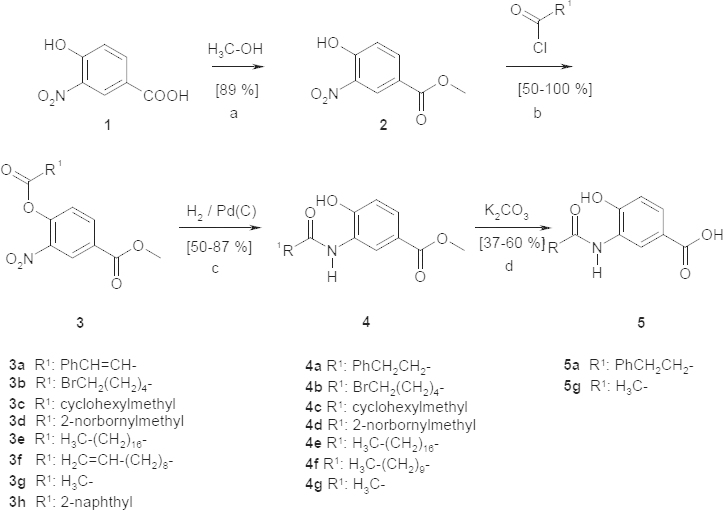
Synthesis of benzoic acid derivatives, a: methanol; b: toluene; c: methanol; d: methanol

Exemplarily, two of the methyl esters were hydrolyzed to give the benzoic acid derivatives **5a** and **5g** as found in the natural products platencin (**A**) and platensimycin (**B**).

In the second series, 2-nitroresorcinol (**6**) was esterified with a half equivalent of acyl chloride to give the monoesters **7a**–**h**. As a byproduct, a remarkable amount of the diesters was observed even when using an excess of 2-nitroresorcinol (**6**), but the diesters could be separated clearly by preparative flash column chromatography. The resulting esters **7a**–**h** were hydrogenated in the way described above to give the anilides **8a**–**h** in high yields (80–90%).

**Sch. 2.**
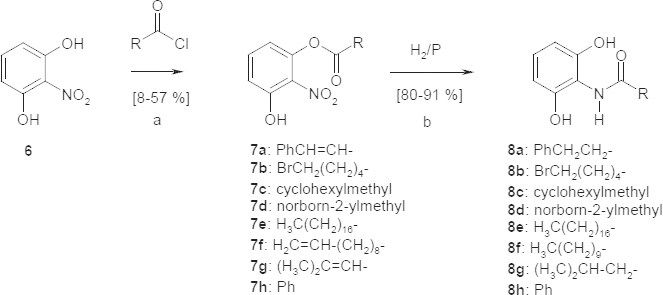
Synthesis of resorcinol derivatives, a: toluene; b: methanol

The mechanism of the aminolysis of the phenol esters is an intramolecular rearrangement as shown by the hydrogenation of an equimolar mixture of **3c** and **7h** and a subsequent GC-MS analysis. The gas chromatogram showed only two peaks of the products **4c** and **8h** and no peak of **8c** or methyl 3-benzamido-4-hydroxybenzoate.

**Tab. 1.** Agar diffusion assay 100 μg / disc, (te: tetracycline, cl: clotrimazol 30 μg/disc); GI (growth inhibition), nt: not tested, zone of inhibition [mm]

	2	3e	4c	4d	4e	4g	5a	8e	8f	te	cl
*Escherichia coli*	25	10 (GI)	0	0	0	0	0	0	0	**25**	**0**
*Pseudomonas antimicrobia*	10	0	0	0	0	0	0	0	0	**23**	**0**
*Staphylococcus equorum*	32	0	0	0	0	0	0	0	**11**	**23**	**9**
*Streptococcus entericus*	20	0	0	0	0	0	0	0	**12**	**12**	**11**
*Candida glabrata*	25 (GI)	10 (GI)	0	0	0	0	0	0	0	nt	**15**
*Aspergillus niger*	22	0	0	0	0	0	0	0	0	nt	**15**
*Yarrowia lipolytica*	12 (GI)	0	0	0	0	0	15 (GI)	0	0	nt	**20**
*Hypopichi burtonii*	23	0	0	0	0	0	0	0	0	nt	**17**

The resulting compounds were tested in an agar diffusion assay [21] against several bacteria (Gram-positive and Gram-negative) and fungi, but showed only weak or no antibiotic activities in this assay as shown exemplarily for some compounds in Table 1. Only the precursor **2** showed an interesting activity against bacteria and fungi.

## Conclusion

The presented synthesis describes a simple and efficient method to prepare *o*-hydroxy-anilides directly from *o*-nitrophenol esters under mild conditions and without affording any protecting group. Thus it is a helpful tool in preparing platensimycin analogues or other natural products containing this partial structure.

The tested compounds themselves showed no or only weak antibiotic activity as shown exemplarily for some compounds (Table 1). This indicates that the *o*-hydroxy-anilide partial structure is not the determining factor alone for the interaction with the FabF enzyme of platencin or platensimycin. The complex cyclic part is also essential for the high antibiotic activity of these natural products.

## Experimental

### General Methods

All solvents used were of HPLC grade or p.a. grade and/or purified according to standard procedures. Chemical reagents were purchased from Sigma Aldrich (Schnelldorf, Germany) and Acros (Geel, Belgium).

IR-spectra: Perkin-Elmer FT-IR Paragon 1000; MS: Hewlett Packard MS-Engine, electron ionisation (EI) 70eV, chemical ionisation (CI) with CH_4_ (300 eV); GC-MS: Shimadzu GC17A, carrier gas: He, column: fused silica capillary column 30 m, detector: EI (70eV). NMR (400 MHz): Jeol GSX 400 (^1^H: 400 MHz, ^13^C: 125 MHz); melting points: Büchi Melting Point B-540 (not corrected); flash column chromatography (FCC): silica gel 60 (230–400 mesh, E. Merck, Darmstadt).

### General Procedure 1 (Preparation of Phenol Esters)

One mmol to 2.0 mmol of the acid were dissolved in 20 mL dry toluene or dry 1,2-dimethoxyethane and 1.0 mL to 2.0 mL (11.5 mmol to 27.5 mmol) SOCl_2_ were added. The solution was refluxed for 1 h, the solvent was evaporated, and the residue was dissolved in 25 mL toluene or 1,2-dimethoxyethane. Alternatively, 0.5 to 1.5 mmol of the commercially available acid chlorides were used. One mmol of the phenol or 1 mmol of the 2-nitroresorcinol and 5 mL *N*-ethyl-*N*-methyl-ethanamine or 5 mL pyridine were added and the solution was stirred for 12 h at room temperature. The solvent was evaporated and the residue was taken up in 30 mL water (for the methyl esters in 30 mL 10% aqueous NaOH) and 30 mL ethyl acetate or diethyl ether. The organic layer was separated and the aqueous layer was again extracted with 30 mL ethyl acetate or diethyl ether, the combined organic layers were dried over Na_2_SO_4_, the solvent was evaporated and the residue was purified by flash column chromatography (isohexane/ethyl acetate 8–10:1).

Alternatively, the reaction could be carried out in a microwave reactor at 80°C for 15 minutes and 235 W, but in most cases with lower yields.

### General Procedure 2 (Hydrogenation)

One mmol of the phenol ester was dissolved in 30 mL methanol and 50 mg 5% Pd on charcoal were added. The suspension was stirred for 14 h under H_2_ atmosphere at room temperature, the catalyst was filtered off (over silica gel 60), and the solvent was evaporated. If necessary, the residue was purified by flash column chromatography.

### General Procedure 3 (Ester Hydrolysis)

An amount of 0.5 mmol of the ester were dissolved in 30 mL methanolic K_2_CO_3_ solution (5%) and refluxed for 24 h. The solvent was evaporated, the residue dissolved in aqueous HCl (10%), and extracted with ethyl acetate (3 × 30 mL). The combined organic layers were dried over Na_2_SO_4_ and the solvent was evaporated. If necessary, the residue was purified by flash column chromatography.

### Methyl (E)-4-[(clnnamoyl)oxy]-3-nltrobenzoate (3a)

The compound was prepared from 230 mg (1.55 mmol) cinnamic acid and 200 mg of **2** (1.01 mmol) according to “General Procedure 1” to give 155 mg (47%) as a pale yellow oil. IR (NaCl, film), v, cm^-1^: 3063, 2951, 2359, 2329, 1724, 1633, 1615, 1537, 1315, 1288, 1188, 1111, 981, 763, 702. MS (CI, m/z, %): 328 (M^+^+1, 2), 131 (100). MS (EI, m/z, %): 131 (100), 103 (36), 77 (18). HR-MS: Calcd. for C_17_H_13_NO_6_: 327.0743 ^g^/_mol_. Found: 327.0731 ^g^/_mol_. ^1^H-NMR (400 MHz, CDCl_3_, TMS): δ 3.97 (s, 3 H, OCH_3_), 6.55 (m, 2 H, aromat. CH, -CH=), 7.43 (m, 3 H, 2 aromat. CH), 7.60 (m, 3 H, 3 aromat. CH), 7.87 (m, 1 H, -CH=), 8.32 (dd, *J* = 2.1 Hz, *J* = 8.4 Hz, 1 H, 6-H), 8.73 (d, *J* = 2.1 Hz, 1 H, 2-H). ^13^C-NMR (125 MHz, CDCl3, TMS): δ 52.9 (OCH_3_), 115.4 (-CH=), 125.6 (aromat. CH), 127.1 (aromat. CH), 128.6 (2 aromat. CH), 129.1 (2 aromat. CH), 131.3 (aromat. CH), 133.7 (quat. C), 135.3 (aromat. CH), 141.9 (quat. C), 147.4 (quat. C), 148.9 (=CH-), 162.5 (quat. C), 163.8 (CO), 164.7 (CO).

### Methyl 4-[(6-bromohexanoyl)oxy]-3-nltrobenzoate (3b)

The compound was prepared according to “General Procedure 1” from 276 mg (1.5 mmol) 6-bromohexanoic acid and 197 mg (1.0 mmol) of **2** to give 306 mg (82%) of **3g** as a pale yellow solid. IR (NaCl, film), v, cm^-1^: 2952, 2867, 1775, 1731, 1616, 1540, 1351, 1288, 1254, 1102. HR-MS: Calcd. for C_14_H_16_BrNO_6_: 373.0161 ^g^/_mol_. Found: 373.0155 ^g^/_mol_. MS (CI, m/z, %): 179 (C_6_H_11_BrO, 42), 177 (C_6_H_11_BrO, 40), 115 (100). ^1^H-NMR (400 MHz, CDCl3, TMS): 1.60 (m, 2 H, CH_2_), 1.82 (tt, *J* = 7.4 Hz, *J* = 7.4 Hz, 2 H, CH_2_), 1.94 (tt, *J* = 6.8 Hz, *J* = 6.9 Hz, 2 H, CH_2_), 2.70 (t, *J* = 7.4 Hz, 2 H, CH_2_), 3.45 (t, *J* = 6.8 Hz, 2 H, CH_2_), 3.98 (s, 3 H, OCH_3_), 7.34 (d, *J* = 8.5 Hz, 1 H, 5-H), 8.32 (dd, *J* = 2.1 Hz, *J* = 8.5 Hz, 1 H, 6-H), 8.75 (d, *J* = 2.1 Hz, 1 H, 2-H). ^13^C-NMR (125 MHz, CDCl_3_, TMS): 23.6 (CH_2_), 27.4 (CH_2_), 32.2 (CH_2_), 33.4 (CH_2_), 33.7 (CH_2_), 52.9 (OCH_3_), 125.5 (aromat. CH), 127.2 (aromat. CH), 128.8 (quat. C), 135.4 (aromat. CH), 141.6 (quat. C), 147.3 (quat. C), 164.4 (CO), 170.5 (CO).

### Methyl 4-[(cyclohexylacetyl)oxy]-3-nltrobenzoate (3c)

The compound was prepared according to “General Procedure 1” from 628 mg (4.42 mmol) cyclohexyl ethanoic acid and 800 mg (4.42 mmol) of **2** to give 1.215 g (86%) of **3c** as a pale yellow solid of mp 40°C. IR (NaCl, film), v, cm^-1^: 2926, 2853, 1775, 1732, 1617, 1541, 1448, 1437, 1352, 1288, 1253, 1097, 924, 823, 769. HR-MS: Calcd. for C_16_H_19_NO_6_: 321.1212 ^g^/_mol_. Found: 321.1213 ^g^/_mol_. MS (El, m/z, %): 321 (M^+^, 0.18), 290 (0.24), 197 (3.6), 181 (4), 166 (13), 125 (100). ^1^H-NMR (400 MHz, CDCl_3_, TMS): 1.00–1.89 (m, 10 H, 5 CH_2_), 1.94 (m, 1 H, CH), 2.54 (d, *J* = 6.9 Hz, 2 H, CH_2_), 3.98 (s, 3 H, CH_3_), 7.32 (d, J = 8.4 Hz, 1 H, aromat. CH), 8.30 (dd, *J* = 2.0 Hz, *J* = 8.4 Hz, 1 H, aromat. CH), 8.72 (d, *J* = 2.0 Hz, 1 H, aromat. CH). ^13^C-NMR (500 MHz, CDCl_3_, TMS): 25.9 (2 CH_2_), 26.0 (CH_2_), 33.0 (2 CH_2_), 34.4 (CH), 41.6 (CH_2_), 52.9 (OCH_3_), 125.60 (aromat. CH), 127.1 (aromat. CH), 128.7 (quat. C), 135.29 (aromat. CH), 141.8 (quat. C), 147.4 (quat. C), 164.4 (CO), 170.1 (CO).

### Methyl 4-[(bicyclo[2.2.1]hept-2-ylacetyl)oxy]-3-nitrobenzoate (3d)

The compound was prepared according to “General Procedure 1” from 230 mg (1.5 mmol) 2-norbornylethanoic acid and 197 mg (1 mmol) of **2** to give 270 mg (81%) of **3d** as a pale yellow solid. IR (NaCl, film), v, cm^-1^: 2952, 2871, 1777, 1731, 1616, 1540, 1437, 1540, 1437, 1351, 1288, 1254, 1091, 918. HR-MS: Calcd. for C_17_H_19_NO_6_: 333.1212 ^g^/_mol_. Found: 333.1213 ^g^/_mol_. MS (El, m/z, %): 333 (M^+^, 0.2), 181 (3), 166 (12), 137 (89), 109 (100). ^1^H-NMR (400 MHz, CDCl_3_, TMS): 1.26–1.54 (m, 8 H, 4 CH_2_), 2.04 (m, 1 H, CH), 2.11 (m, 1 H, CH), 2.28 (m, 1 H, CH), 2.49 (dd, *J* = 7.8 Hz, *J* = 16.1 Hz, 1 H, CH_2_), 2.64 (dd, *J* = 7.7 Hz, *J* = 16.1 Hz, 1 H, CH_2_), 3.98 (s, 3 H, OCH_3_), 7.32 (d, *J* = 8.4 Hz, 1 H, 5-H), 8.31 (ddd, *J* = 0.9 Hz, *J* = 2.0 Hz, *J* = 8.4 Hz, 1 H, 6-H), 8.74 (d, *J* = 2.0 Hz, 1 H, 2-H). ^13^C-NMR (125 MHz, CDCl_3_, TMS): 28.6 (CH_2_), 29.8 (CH_2_), 35.4 (CH_2_), 36.9 (CH), 37.9 (CH_2_), 38.0 (CH), 41.0 (CH_2_); 41.3 (CH), 53.0 (CH_3_), 125.7 (aromat. CH), 127.3 (aromat. CH), 128.7 (quat. C), 135.4 (aromat. CH), 141.7 (quat. C), 147.4 (quat. C), 164.4 (CO), 170.2 (CO).

### Methyl 3-nltro-4-(octadecanoyloxy)benzoate (3e)

The compound was prepared according to “General Procedure 1” from 620 mg (2.0 mmol) octadecanoyl chloride and 300 mg (1.5 mmol) of **2** to give 730 mg (100%) of **3e** as a white solid of mp 70°C. HR-MS: Calcd. for C_26_H_41_NO_6_: 463.293389 ^g^/_mol_. Found: 463.2932 ^g^/_mol_. MS (El, m/z, %): 463 (M^+^, 0,12), 284 (8), 267 (100), 181 (48), 166 (32). ^1^H-NMR (400 MHz, CDCl_3_, TMS): 0.88 (t, *J* = 7.0 Hz, 3 H, CH_3_), 1.26 (m, 26 H, 13 CH_2_), 1.42 (m, 2 H, CH_2_), 1.65 (m, 2 H, CH_2_), 1.77 (m, 2 H, CH_2_), 2.66 (t, *J* = 7.6 Hz, 2 H, CH_2_), 3.98 (s, 3 H, OCH_3_), 7.33 (d, *J* = 8.5 Hz, 1 H, aromat. CH), 8.31 (dd, *J* = 2.1 Hz, *J* = 8.5 Hz, 1 H, 2-H), 8.74 (d, *J* = 2.1 Hz, 1 H, aromat. CH). ^13^C-NMR (125 MHz, CDCl_3_, TMS): 14.2 (CH_3_), 22.7 (CH_2_), 24.4 (CH_2_), 29.2 (CH_2_), 29.4 (CH_2_), 29.4 (CH_2_), 29.6 (CH_2_), 29.7 (CH_2_), 29.7 (CH_2_), 29.7 (CH_2_), 29.7 (5 CH_2_), 31.9 (CH_2_), 34.0 (CH_2_), 52.9 (CH_3_O), 125.6 (aromat. CH), 127.2 (aromat. CH), 128.8 (quat. C), 135.4 (aromat. CH), 135.4 (quat. C), 147.5 (quat. C), 164.4 (CO), 171.0 (CO).

### Methyl 3-nltro-4-(undec-10-enoyloxy)benzoate (3f)

The compound was prepared according to “General Procedure 1” from 500 mg (2.7 mmol) undec-10-enoic acid and 355 mg (1.8 mmol) of **2** to give 425 mg (65%) of **3f** as a pale yellow solid. MS (CI, m/z, %): 364 (M^+^+1, 2), 167 (100). ^1^H-NMR (400 MHz, CDCl_3_, TMS): δ 1.29 (m, 10 H, 5 CH_2_), 1.63 (m, 2 H, CH_2_), 2.04 (m, 2 H, CH_2_), 2.35 (t, *J* = 7.5 Hz, 2 H, CH_2_), 3.95 (s, 3 H, OCH_3_), 4.96 (m, 2 H, =CH_2_), 5.81 (ddt, *J* = 6.7 Hz, *J* = 10.4 Hz, *J* = 17.0 Hz, 1 H, =CH-), 7.23 (d, *J* = 8.8 Hz, 1 H, aromat. CH), 8.24 (dd, J = 2.1 Hz, *J* = 8.8 Hz, 1 H, aromat. CH), 8.83 (d, *J* = 2.1 Hz, 1 H, aromat. CH). ^13^C-NMR (125 MHz, CDCl_3_, TMS): 24.6 (CH_2_), 28.8 (CH_2_), 29.0 (CH_2_), 29.0 (2 CH_2_), 29.2 (CH_2_), 29.2 (CH_2_), 33.8 (CH_2_), 33.9 (CH_2_), 52.6 (OCH_3_), 114.1 (=CH_2_), 120.2 (aromat. CH), 127.3 (aromat. CH), 137.9 (aromat. CH), 139.2 (-CH=), 158.1 (quat. C), 164.8 (CO), 179.5 (CO).

### Methyl 4-(acetyloxy)-3-nitrobenzoate (3g)

The compound was prepared according to “General Procedure 1” from 478 mg (2.0 mmol) of **2** and 234 mg (3.0 mmol) acetyl chloride to give 185 mg (41%) of **3g** as a pale yellow oil. IR (KBr), v, cm^-1^:3269, 2955, 1724, 1626, 1583, 1540, 1435, 1329, 1288, 1182, 1143, 760. HR-MS: Calcd. for C_10_H_9_NO_6_: 239.0430 ^g^/_mol_. Found: 239.0428 ^g^/_mol_. MS (CI, m/z, %): 240 (M^+^+1, 30), 198 (100). MS (El, m/z, %): 239 (M^+^, 2), 197 (70), 166 (100), 120 (26), 63 (28). ^1^H-NMR (400 MHz, CDCl_3_, TMS): δ 2.41 (s, 3 H, CH_3_), 3.98 (s, 3 H, OCH_3_), 7.34 (d, *J* = 8.7 Hz, 1 H, aromat. CH), 8.32 (dd, *J* = 2.2 Hz, *J* = 8.5 Hz, 1 H, aromat. CH), 8.75 (d, *J* = 2.2 Hz, 1 H, aromat. CH). ^13^C-NMR (125 MHz, CDCl_3_, TMS): 20.76 (CH3), 52.84 (OCH_3_), 125.55 (aromat. CH), 127.2 (aromat. CH), 128.9 (quat. C), 135.4 (aromat. CH), 147.3 (quat. C), 164.3 (CO), 168.0 (CO).

### 4-(Methoxycarbonyl)-2-nitrophenyl naphthalene-2-carboxylate (3h)

The compound was prepared according to “General Procedure 1” from 300 mg (1.57 mmol) 2-naphthoyl chloride and 210 mg (1.06 mmol) **2** to give 506 mg (92%) of **3h** as colourless crystals of mp 144°C. IR (KBr), v, cm^-1^: 1737, 1612, 1537, 1280, 1255, 1222, 1193, 1046, 757. MS (CI, m/z, %): = 352 (M^+^+1, 2), 173 (14), 155 (100). MS (El, m/z, %): 351 (M^+^, 4), 326 (8), 155 (100), 127 (52). HR-MS: Calcd for C_19_H_13_NO_6_: 351.0743 ^g^/_mol_. Found: 351.0757 ^g^/_mol_. ^1^H-NMR (400 MHz, CDCl_3_, TMS): δ 4.00 (s, 3 H, CH_3_), 7.55 (d, *J* = 8.2 Hz, 1 H, CH), 7.60 (dd, *J* = 8.2 Hz, *J* = 8.2 Hz, 1 H, CH), 7.66 (dd, *J* = 8.2 Hz, *J* = 8.2 Hz, 1 H, CH), 7.93 (d, *J* = 8.2 Hz, 1 H, CH), 7.97 (d, *J* = 8.2 Hz, 1 H, CH), 8.02 (d, *J* = 8.2 Hz, 1 H, CH), 8.16 (dd, *J* = 1.8 Hz, *J* = 8.2 Hz, 1 H, CH), 8.38 (dd, *J* = 1.8 Hz, *J* = 8.2 Hz, 1 H, CH), 8.48 (m, 2 H, 2 aromat. CH). ^13^C-NMR (125 MHz, CDCl_3_, TMS): 52.87 (CH_3_), 125.3 (aromat. CH), 125.7 (aromat. CH), 125.7 (quat. C), 127.0 (aromat. CH), 127.3 (aromat. CH) 127.9 (aromat. CH), 128.7 (CH), 128.7 (quat. arom. C), 129.1 (CH), 129.6 (CH), 132.4 (quat. arom. C), 132.9 (aromat. CH), 135.4 (aromat. CH), 136.2 (quat. C), 140.0 (quat. C), 147.7 (quat. C), 164.1 (CO), 164.4 (CO).

### Methyl 4-hydroxy-3-[(3-phenylpropanoyl)amlno]benzoate (4a)

The compound was prepared according to “General Procedure 2” from 150 mg (0.46 mmol) **3a** to give 120 mg (87%) of **4a** as colourless crystals of mp 152°C. IR (NaCl, film), v, cm^-1^: 3412, 3026, 2958, 2720, 1899, 1708, 1654, 1595, 1543, 1450, 1437, 1293, 1275, 1126, 766, 697, 631. HR-MS: Calcd. for C_17_H_17_NO_4_: 299.1158. Found: 299.1162. MS (CI, m/z, %): 300 (M^+^+1, 100), 167 (12). MS (El, m/z, %): 299 (M^+^, 10), 167 (100), 136 (22), 105 (24), 91 (46). ^1^H-NMR (400 MHz, CDCl_3_, TMS): δ 2.78 (t, *J* = 7.5 Hz, 2 H, CH_2_), 3.07 (t, *J* = 7.5 Hz, 2 H, CH_2_), 3.83 (s, 3 H, OCH_3_), 6.99 (d, *J* = 8.7 Hz, 1 H, aromat. CH), 7.24 (m, 3 H, 3 aromat. CH), 7.31 (m, 2 H, 2 aromat. CH), 7.76 (m, 2 H, 2 aromat. CH), 8.16 (s, 1 H, NH), 10.02 (s, 1 H, OH). ^13^C-NMR (125 MHz, CDCl_3_, TMS): 31.7 (CH_2_), 38.7 (CH_2_), 52.1 (OCH_3_), 119.9 (aromat. CH), 122.0 (quat. C), 124.2 (aromat. CH), 125.5 (quat. C), 126.8 (aromat. CH), 128.3 (2 aromat. CH), 128.9 (aromat. CH), 128.9 (2 aromat. CH), 139.8 (quat. C), 153.4 (quat. C), 166.6 (CO), 173.3 (CO).

### Methyl 3-[(6-bromohexanoyl)amlno]-4-hydroxybenzoate (4b)

The compound was prepared according to “General Procedure 2” from **330** mg (0.88 mmol) **3b** to give 150 mg (50%) of **4b** as a pale brown solid of mp 132°C. IR (KBr), v, cm^-1^: 3406, 3066, 2954, 2856, 1703, 1667, 1610, 1593, 1433, 1447, 1433, 1273, 1125, 997, 908, 770, 642, 631. HR-MS: Calcd. for C_14_H_18_BrNO_4_: 343.0419. Found: 343.0392. MS (EI, m/z, %): 345 (M^+^, 0.75), 343 (M^+^, 0.74), 167 (100), 136 (19). ^1^H-NMR (400 MHz, d-DMSO, TMS): δ 1.42 (m, 2 H, CH_2_), 1.60 (m, 2 H, CH_2_), 1.84 (m, 2 H, CH_2_), 2.42 (t, *J* = 7.6 Hz, 2 H, CH_2_), 3.55 (t, *J* = 6.7 Hz, 2 H, CH_2_), 3.79 (s, 3 H, OCH_3_), 6.94 (d, *J* = 8.5 Hz, 1 H, 5-H), 7.58 (dd, *J* = 2.0 Hz, *J* = 8.5 Hz, 1 H, 6-H), 8.52 (s, 1 H, 2-H), 9.28 (s, 1 H, NH), 10.83 (s, 1 H, OH). ^13^C-NMR (125 MHz, d-DMSO, TMS): 22.50 (CH_2_), 26.5 (CH_2_), 27.8 (CH_2_), 32.6 (CH_2_), 35.7 (CH_2_), 51.7 (OCH_3_), 115.1 (aromat.CH), 120.14 (quat. C), 123.3 (aromat. CH), 126.2 (aromat. CH), 126.4 (quat. C), 152.2 (quat. C), 166.1 (CO), 171.8 (CO).

### Methyl 3-[(cyclohexylacetyl)amino]-4-hydroxybenzoate (4c)

The compound was prepared according to “General Procedure 2” from 211 mg (0.66 mmol) **3c** to give 171 mg (89%) of **4c** as a pale yellow solid of mp 165°C. IR (KBr), v, cm^-1^: 3288, 2922, 2851, 1712, 1623, 1591, 1549, 1450, 1297, 1234, 1131, 764. HR-MS: Calcd. for C_16_H_21_NO_4_: 291.1471. Found: 291.1500. MS (EI) m/z (%) = 291 (10), 167 (100). ^1^H-NMR (400 MHz, D_3_COD, TMS): 0.98-1.89 (m, 11 H, CH, 5 CH_2_), 2.31 (d, *J* = 7.1 Hz, 2 H, CH_2_), 3.84 (s, 3 H, OCH_3_), 6.89 (d, *J* = 8.5 Hz, 1 H, 5-H), 7.68 (dd, *J* = 2.2 Hz, *J* = 8.5 Hz, 1 H, 6-H), 8.41 (d, *J* = 2.2 Hz, 1 H, 2-H), 9.23 (s, 1 H, NH, only in d-DMSO), 10.93 (s, 1 H, OH, only in d-DMSO). ^13^C-NMR (125 MHz, D_3_COD, TMS): 27.3 (2 CH_2_), 27.30 (CH_2_), 34.1 (2 CH_2_), 37.0 (CH), 45.6 (CH_2_), 52.3 (OCH_3_), 116.6 (aromat. CH), 122.0 (quat. C), 125.5 (aromat. CH), 127.2 (quat. C), 128.6 (aromat. CH), 154.8 (quat. C), 168.5 (CO), 174.5 (CO).

### Methyl 3-[(blcyclo[2.2.1]hept-2-ylacetyl)amlno]-4-hydroxybenzoate (4d)

The compound was prepared according to “General Procedure 2” from 335 mg (1.0 mmol) **3d** to give 257 mg (77%) of **4d** as a pale yellow solid of mp 146°C. IR (KBr), v, cm^-1^: 3407, 2949, 2867, 1708, 1660, 1611, 1595, 1541, 1449, 1434, 1288, 1280, 1262, 1125, 767. HR-MS: Calcd. for C_17_H_21_NO_4_: 303.147059. Found: 303.1478. MS (EI) m/z (%) = 303 (M^+^, 8), 167 (100), 136 (16), 112 (20). ^1^H-NMR (400 MHz, d-acetone, TMS): 1.02-1.58 (m, 10 H, 2 CH, 4 CH_2_), 1.96-2.23 (m, 1 H, CH_2_), 2.35 (dd, *J* = 7.9 Hz, *J* = 14.3 Hz, 1 H, CH_2_), 2.48 (dd, *J* = 7.9 Hz, *J* = 14.4 Hz, 1 H, CH_2_), 3.81 (s, 3 H, OCH_3_), 6.95 (d, *J* = 8.4 Hz, 1 H, 5-H), 7.65 (dd, *J* = 2.0 Hz, *J* = 8.4 Hz, 1 H, 6-H), 8.42 (d, *J* = 2.0 Hz, 1 H, 2-H), 9.17 (s, 1 H, OH). ^13^C-NMR (125 MHz, d-acetone, TMS): 29.1 (CH_2_), 30.3 (CH_2_), 35.5 (CH_2_), 37.3 (CH), 38.4 (CH_2_), 39.7 CH), 41.6 (CH), 43.9 (CH_2_), 51.7 (OCH_3_), 117.4 (aromat. CH), 121.2 (quat. C), 123.3 (aromat. CH), 127.5 (aromat. CH), 127.6 (quat. C), 154.0 (quat. C), 166.8 (CO), 172.8 (CO).

### Methyl 4-hydroxy-3-(octadecanoylamlno)benzoate (4e)

The compound was prepared according to “General Procedure 2” from 390 mg (0.84 mmol) **3e** to give 190 mg (52%) of **4e** as a pale brown solid. MS (CI, m/z, %): 434 (M^+^+1, 54), 326 (74), 312 (100), 285 (46), 267 (46), 168 (98). MS (El, m/z, %): 433 (M^+^, 4), 167 (100). ^1^H-NMR (400 MHz, d-DMSO, TMS): δ 0.86 (t, *J* = 7.2 Hz, CH_3_), 1.24 (m, 28 H, 14 CH_2_), 1.59 (m, 2 H, CH_2_), 2.40 (t, *J* = 7.5 Hz, 2 H, CH_2_), 3.80 (s, 3 H, OCH_3_), 4.08 (s, 1 H, NH), 6.94 (d, *J* = 8.5 Hz, 1 H, aromat. CH), 7.59 (dd, *J* = 2.0 Hz, *J* = 8.5 Hz, 1 H, aromat. CH), 8.49 (d, *J* = 2.0 Hz, 1 H, aromat. CH). ^13^C-NMR (125 MHz, d-DMSO, TMS): 14.0 (CH_3_), 22.3 (CH_2_), 25.4 (CH_2_), 28.8 (CH_2_), 28.9 (CH_2_), 29.0 (CH_2_), 29.1 (CH_2_), 29.2 (CH_2_), 29.2 (CH_2_), 29.2 (CH_2_), 29.24 (5 CH_2_), 31.49 (CH_2_), 36.14 (CH_2_), 51.6 (OCH_3_), 115.3 (aromat. CH), 120.6 (quat. C), 123.3 (aromat. CH), 126.3 (aromat. CH), 152.0 (quat. C), 166.2 (CO), 172.2 (CO).

### Methyl 4-hydroxy-3-(undecanoylamino)benzoate (41)

The compound was prepared according to “General Procedure 2” from 250 mg (0.69 mmol) **3e** to give 190 mg (82%) of **4e** as a pale yellow solid. HR-MS: Calcd. for C_19_H_29_NO_4_: 335.209659 ^g^/_mol_. Found: 335.2097 ^g^/_mol_. MS (CI, m/z, %): 336 (M^+^+1, 100), 167 (20). MS (EI, m/z, %): 335 (M^+^, 2), 167 (100). ^1^H-NMR (400 MHz, CDCl_3_, TMS): δ 0.86 (t, *J* = 6.7 Hz, 3 H, CH_3_), 1.23 (m, 14 H, 7 CH_2_), 1.61 (m, 2 H, CH_2_), 2.32 (t, *J* = 7.4 Hz, 2 H, CH_2_), 3.84 (s, 3 H, OCH_3_), 6.98 (d, *J* = 8.5 Hz, 1 H, aromat. CH), 7.74 (dd, *J* = 2.0 Hz, *J* = 8.5 Hz, 1 H, aromat. CH), 7.85 (d, *J* = 2.0 Hz, 1 H, aromat. CH), 8.32 (s, 1 H, NH). ^13^C-NMR (125 MHz, CDCl_3_, TMS): 14.1 (CH_3_), 22.6 (CH_2_), 24.8 (CH_2_), 25.8 (CH_2_), 29.2 (CH_2_), 29.3 (CH_2_), 29.4 (CH_2_), 31.8 (CH_2_), 33.99 (CH_2_), 36.8 (CH_2_), 52.0 (OCH_3_), 119.2 (aromat. CH), 124.0 (aromat. CH), 125.8 (quat. C), 128.4 (aromat. CH), 153.1 (quat. C), 166.8 (quat. C), 174.4 (CO), 179.5 (CO).

### Methyl 3-(acetylamlno)-4-hydroxybenzoate (4g)

The compound was prepared according to “General Procedure 2” from 900 mg (4.56 mmol) **3g** to give 710 mg (74%) of **4d** as pale brown crystals of mp 176°C. IR (NaCl, film), v, cm^-1^: 3278, 3092, 2965, 2721, 1907, 1714, 1592, 1554, 1504, 1425, 1382, 1282, 1231, 1129, 1095, 988, 902, 832, 763. HR-MS: Calcd. for C_10_H_11_NO_4_: 209.0688 ^g^/_mol_ Found: 209.0667 ^g^/_mol_. MS (CI, m/z, %): 210 (M^+^+1, 100). MS (EI) m/z (%) = 193 (14), 167 (32), 79 (32). ^1^H-NMR (400 MHz, CDCl_3_, TMS): δ 2.10 (s, 3 H, COCH_3_), 3.78 (s, 3 H, OCH_3_), 6.94 (d, *J* = 8.4 Hz, 1 H, CH), 7.58 (dd, *J* = 2.0 Hz, *J* = 8.4 Hz, 1 H, CH), 8.48 (d, *J* = 2.0 Hz, 1 H, CH), 9.32 (s, 1 H, NH), 10.81 (s, 1 H, OH). ^13^C-NMR (125 MHz, d-DMSO, TMS): δ (ppm) = 23.6 (CH_3_), 51.6 (OCH_3_), 116.0 (aromat. CH), 120.0 (quat. C), 123.2 (aromat. CH), 126.1 (aromat. CH), 126.2 (quat. C), 152.1 (quat. C), 166.0 (CO), 168.9 (CO). The compound is also described in the literature [19].

### 4-Hydroxy-3-[(3-phenylpropanoyl)amino]benzoic acid (5a)

The compound was prepared according to “General Procedure 3” from 140 mg (0.46 mmol) of **4a** to give 48 mg (37%) of **5a** as a pale yellow oil. IR (NaCl, film), v, cm^-1^: 3423, 2360, 2342, 1709, 1665, 1597, 1547, 1439, 1383, 1274, 784, 768, 668. MS (CI, m/z, %): 286 (M^+^+1, 4), 272 (12), 165 (20), 151 (100), 139 (88), 133 (94), 120 (58), 102 (62). MS (El, m/z, %): 285 (M^+^, 2), 167 (14), 139 (22), 104 (26), 91 (100). ^1^H-NMR (400 MHz, CDCl_3_, TMS): δ 2.72 (t, *J* = 7.7 Hz, 2 H, CH_2_), 2.90 (t, *J* = 7.7 Hz, 2 H, CH_2_), 6.90 (d, *J* = 8.0 Hz, 1 H, 5-H), 7.19 (m, 3 H, 3 aromat. CH), 7.28 (m, 2 H, 2 aromat. CH), 7.53 (dd, *J* = 2.0 Hz *J* = 8.0 Hz, 1 H, 6-H), 8.43 (d, *J* = 2.1 Hz, 1 H, 2-H), 9.34 (s, 1 H, OH). ^13^C-NMR (125 MHz, CDCl_3_, TMS): 30.9 (CH_2_), 37.4 (CH_2_), 114.9 (aromat. CH), 123.3 (aromat. CH), 125.8 (aromat. CH), 126.2 (aromat. CH), 128.1 (2 aromat. CH), 128.2 (2 aromat. CH), 140.8 (quat. C), 141.1 (quat. C), 152.2 (quat. C), 167.4 (quat. C), 170.8 (CO), 173.8 (CO).

### 3-(Acetylamlno)-4-hydroxybenzolc acid (5g)

The compound was prepared according to “General Procedure 3” from 113 mg (0.54 mmol) of **4d** to give 63 mg (60%) of **5d** as a brown solid. Mp 239°C. IR (KBr, film), v, cm^-1^: 3423, 2964, 1673, 1596, 1543, 1458, 1411, 1282, 1242, 1123, 1098, 969, 836, 629, 585, 549. MS (EI, m/z, %): 195 (M+, 20), 153 (100), 136 (28), 109 (50), 80 (16). HR-MS: Calcd. for C_9_H_9_NO_4_: 195.053159 ^g^/_mol_. Found: 195.0535 ^g^/_mol_. ^1^H-NMR (400 MHz, d-DMSO, TMS): δ 2.09 (s, 3 H, CH_3_), 6.90 (d, *J* = 8.4 Hz, 1 H, 5-H), 7.54 (dd, *J* = 2.0 Hz, *J* = 8.4 Hz, 1 H, 6-H), 8.40 (d, *J* = 2.0 Hz, 1 H, 2-H), 9.28 (s, 1 H, NH), 10.64 (s, 1 H, OH), 12.42 (s, 1 H, COOH). ^13^C-NMR (125 MHz, CDCl_3_, TMS): 23.7 (CH_3_), 115.0 (aromat. CH), 121.3 (quat. C), 123.7 (aromat. CH), 126.2 (quat. C), 126.4 (aromat. CH), 151.9 (quat. C), 167.2 (CO), 169.0 (CO). The compound is described in the literature [20].

### 3-Hydroxy-2-nitrophenyl (E)-cinnamate (7a)

The compound was prepared according “General Procedure 2” (MW conditions) from 296 mg (2 mmol) cinnamic acid and 465 mg (3 mmol) 2-nitroresorcinol to give 215 mg (38%) of **7a** as a yellow solid of mp 95°C. IR (NaCl, film), v, cm^-1^: 3425, 1740, 1636, 1613, 1585, 1541, 1449, 1346, 1221, 1196, 119, 1033, 762. ESI-MS (CI, m/z, %): 283 (10), 2 (100), 131 (15). ^1^H-NMR (400 MHz, CDCl_3_, TMS): δ 6.67 (d, *J* = 15.7 Hz, 1 H, -CH=), 6.81 (dd, *J* = 1.2 Hz, *J* = 8.1 Hz, 1 H, aromat. CH), 7.09 (dd, *J* = 1.2 Hz, *J* = 8.6 Hz, 1H, aromat. CH), 7.44 (m, 3 H, 3 aromat. CH), 7.53 (dd, *J* = 8.1 Hz, *J* = 8.6 Hz, 1 H, aromat. CH), 7.61 (m, 2 H, 2 aromat. CH), 7.91 (d, *J* = 15.7 Hz, 1 H, -CH=), 10.39 (s, 1 H, OH). ^13^C-NMR (125 MHz, CDCl_3_, TMS): 115.9 (CH), 115.9 (CH), 117.4 (CH), 128.5 (2 aromat. CH), 128.6 (quat. C), 129.0 (2 aromat. CH), 131.1 (aromat. CH), 133.8 (quat. C), 135.5 (aromat. CH), 145.5 (quat. C), 148.2 (-CH=), 155.5 (quat. C), 164.5 (CO).

### 3-Hydroxy-2-nitrophenyl 6-bromohexanoate (7b)

The compound was prepared according to “General Procedure 1” from 0.982 g (5.0 mmol) 6-bromohexanoic acid and 1.53 g (9.9 mmol) 2-nitroresorcinol to give 0.624 g (37%) of **7b**. IR (KBr), v, cm^-1^: 3374, 2940, 2866, 1771, 1616, 1586, 1541, 1461, 1353, 1190, 1108, 1056, 858, 808, 688. MS (EI, m/z, %): 179 (18), 177 (20), 155 (10), 97 (24), 69 (100), 60 (63), 55 (52). MS (CI, m/z, %): 179 (78), 177 (78), 122 (25), 115 (100). HR-MS: Calcd. for C_12_H_14_BrNO_5_: 331.0055 ^g^/_mol_. Found: 331.0052 ^g^/_mol_. ^1^H-NMR (400 MHz, CDCl_3_, TMS): δ 1.60 (m, 2 H, CH_2_), 1.80 (m, 2 H, CH_2_), 1.93 (m, 2 H, CH_2_), 2.68 (t, *J* = 7.7 Hz, 2 H, CH_2_), 3.44 (t, *J* = 6.7 Hz, 2 H, CH_2_), 6.71 (dd, *J* = 1.4 Hz, *J* = 8.0 Hz, 1 H, 4-H), 7.07 (dd, *J***?** = 8.6 Hz, *J* = 1.4 Hz, 1 H, 6-H), 7.51 (dd, *J* = 8.0 Hz, *J* = 8.6 Hz, 1 H, 5-H), 10.40 (s, 1 H, OH). ^13^C-NMR (125 MHz, CDCl_3_, TMS): 23.5 (CH_2_), 27.5 (CH_2_), 32.3 (CH_2_), 33.4 (CH_2_), 33.8 (CH_2_), 115.9 (aromat. CH), 117.6 (aromat. CH), 129.0 (quat. C), 135.9 (aromat. CH), 145.6 (quat. C), 156.0 (quat. C), 171.2 (CO).

### 3-Hydroxy-2-nitrophenyl cyclohexylacetate (7c)

The compound was prepared according to “General Procedure 1” from 0.717 g (5.05 mmol) 2-cyclohexylacetic acid and 0.7810 (5.04 mmol) 2-nitroresorcinol to give 312 mg (20%) of **7c**. IR (NaCl, film), v, cm^-1^: 2925, 2852, 1772, 1616, 1586, 1540, 1462, 1450, 1353, 1211, 1101, 1057, 1027, 872, 858, 808, 687. MS (CI, m/z, %): 157 (9), 143 (23), 125 (100). MS (EI, m/z, %): 279 (M^+^, 1), 155 (4), 139 (3), 125 (100). ^1^H-NMR (400 MHz, CDCl_3_, TMS): δ 1.23 (m, 6 H, 3 CH_2_), 1.68 (m, 4 H, 2 CH_2_), 1.93 (m, 1 H, CH), 2.52 (d, *J* = 7.0 Hz, 2 H, CH_2_), 6.69 (dd, *J* = 1.4 Hz, *J* = 8.0 Hz, 1 H, 4-H), 7.06 (dd, *J* = 1.4 Hz, *J* = 8.6 Hz, 1 H, 6-H), 7.50 (dd, *J* = 8.0 Hz, *J* = 8.6 Hz, 1 H, 5-H), 10.35 (s, 1 H, OH). ^13^C-NMR (125 MHz, CDCl_3_, TMS): 26.0 (2 CH_2_), 26.1 (CH_2_), 33.0 (2 CH_2_), 34.4 (CH), 41.6 (CH_2_), 116.0 (aromat CH), 117.4 (aromat CH), 135.8 (aromat CH), 145.7 (quat. C), 155.9 (quat. C), 170.8 (CO).

### 3-Hydroxy-2-nitrophenyl bicyclo[2.2.1]hept-2-ylacetate (7d)

The compound was prepared according to “General Procedure 1” from 0.783 g (5.6 mmol) norbornylacetic acid and 1.472 g (9.5 mmol) 2-nitroresorcinol to give 0.392 g (27%) of **7d** as a pale yellow solid. IR (KBr), v, cm^-1^: 3356, 2951, 2870, 1773, 1617, 1586, 1541, 1458, 1353, 1208, 1169, 1120, 1103, 1057, 1031, 912, 856, 807, 687. HR-MS: Calcd. for C_15_H_17_NO_5_: 291.1107 ^g^/_mol_ Found: 291.1107 ^g^/_mol_. MS (EI, m/z, %): 291 (M^+^, 8), 137 (100), 109 (90), 95 (60), 67 (62). ^1^H-NMR (400 MHz, CDCl_3_, TMS): δ 1.18-1.62 (m, 9 H, CH, 4 CH_2_), 2.11 (m, 1 H, CH_2_), 2.27 (m, 1 H, CH_2_), 2.47 (dd, *J* = 7.8 Hz, *J* = 16.0 Hz, 1 H, CH_2_), 2.62 (dd, *J* = 7.8 Hz, *J* = 16.0 Hz, 1 H, CH_2_), 6.69 (dd, *J* = 1.4 Hz, *J* = 8.0 Hz, 1 H, 4-H), 7.06 (dd, *J* = 1.4 Hz, *J* = 8.6 Hz, 1 H, 6-H), 7.5 (dd, *J* = 8.0 Hz, *J* = 8.6 Hz, 1 H, 5-H), 10.37 (s, 1 H, OH). ^13^C-NMR (125 MHz, CDCl_3_, TMS): 28.5 (CH_2_), 29.7 (CH_2_), 35.3 (CH_2_), 36.8 (CH), 37.9 (CH_2_), 37.9 (CH), 40.9 (CH_2_), 41.2 (CH), 117.0 (CH), 117.4 (CH), 135.8 (CH), 145.7 (quat. C), 155.9 (quat. C), 170.8 (CO).

### 3-Hydroxy-2-nitrophenyl octadecanoate (7e)

The compound was prepared according to “General Procedure 1” from 302 mg (1 mmol) octadecanoyl chloride and 310 mg (2 mmol) 2-nitroresorcinol to give 75 mg (18%) of **7e** as an orange solid of mp 58°C. IR (KBr), v, cm^-1^: 2954, 2923, 2849, 1764, 1617, 1546, 1469, 1349, 1140, 1122, 1055, 814, 691. HR-MS: Calcd. for C_24_H_38_NO_5_: 420.274999 ^g^/_mol_. Found: 420.2755 ^g^/_mol_. ^1^H-NMR (400 MHz, CDCl_3_, TMS): δ 0.88 (t, *J* = 6.5 Hz, 3 H, CH_3_), 1.26 (m, 28 H, 14 CH_2_), 1.77 (tt, *J* = 7.3 Hz, *J* = 7.3 Hz, 2 H, CH_2_), 2.65 (t, *J* = 7.8 Hz, 2 H, CH_2_), 6.70 (dd, *J* = 1.3 Hz, *J* = 8.0 Hz, 1 H, aromat. CH), 7.07 (dd, *J* = 1.3 Hz, *J* = 8.4 Hz, 1 H, aromat. CH), 7.51 (dd, *J* = 8.0 Hz, *J* = 8.4 Hz, 1 H, aromat. CH), 10.42 (s, 1 H, OH). ^13^C-NMR (125 MHz, CDCl_3_, TMS): 14.1 (CH_3_), 22.7 (CH_2_), 24.4 (CH_2_), 29.1 (CH_2_), 29.2 (CH_2_), 29.4 (CH_2_), 29.4 (CH_2_), 29.6 (CH_2_), 29.6 (CH_2_), 29.7 (CH_2_), 29.7 (5 CH_2_), 31.9 (CH_2_), 34.0 (CH_2_), 115.9 (aromat. CH), 117.5 (aromat. CH), 124.1 (quat. C), 135.8 (aromat. CH), 145.7 (quat. C), 155.9 (quat. C), 171.5 (CO).

### 3-Hydroxy-2-nitrophenyl undec-10-enoate (7f)

The compound was prepared according to “General Procedure 1” from 368 mg (2 mmol) 10-undecenoic acid and 310 mg (2 mmol) 2-nitroresorcinol to give 165 mg (26%) **7f** as a pale yellow oil. IR (KBr), v, cm^-1^: 2927, 2855, 1774, 1640, 1617, 1587, 1541, 1463, 1357, 1196, 1106, 1057, 1030, 910, 857. HR-MS (M^+^-1): Calcd. for C_17_H_22_NO_5_: 320.1498 ^g^/_mol_. Found: 320.1505 ^g^/_mol_. MS (EI, m/z, %): 274 (1), 183 (7), 167 (15), 149 (100), 139 (30), 107 (50). ^1^H-NMR (400 MHz, CDCl_3_, TMS): δ 1.24-1.44 (m, 10 H, 5 CH_2_), 1.73 (m, 2 H, CH_2_), 2.04 (m, 2 H, CH_2_), 2.57 (t, *J* = 7.9 Hz, 2 H, CH_2_), 4.93 (m, 1 H, =CH_2_), 4.99 (m, 1 H, =CH_2_), 5.81 (ddt, *J* = 6.7 Hz, *J* = 10.4 Hz, *J* = 16.9 Hz, 1 H, -CH=), 6.69 (d, *J* = 8.3 Hz, 1 H, aromat. CH), 7.01 (d, *J* = 8.4 Hz, 1 H, aromat. CH), 7.38 (dd, *J* = 8.3 Hz, *J* = 8.3 Hz, 1 H, aromat. CH), 10.49 (s, 1 H, OH). ^13^C-NMR (125 MHz, CDCl_3_, TMS): 24.5 (CH_2_), 28.8 (CH_2_), 28.9 (CH_2_), 29.0 (CH_2_), 29.1 (CH_2_), 29.2 (CH_2_), 33.7 (CH_2_), 33.9 (CH_2_), 114.2 (H_2_C=), 114.7 (aromat. CH), 116.2 (aromat. CH), 131.9 (quat. C), 133.1 (aromat. CH), 139.1 (-CH=), 144.3 (quat. C), 153.1 (quat. C), 171.2 (CO).

### 3-Hydroxy-2-nitrophenyl 3-methylbut-2-enoate (7g)

The compound was prepared according to “General Procedure 1” from 0.515 g (5.2 mmol) 3,3-dimethylacrylic acid and 0.775 g (5 mmol) 2-nitroresorcinol to give 0.095 g (8%) of **7g**. IR (KBr), v, cm^-1^: 3224, 2979, 2935, 1739, 1693, 1649, 1614, 1585, 1537, 1442, 1348, 1115, 1057, 937, 818, 679. MS (CI, m/z, %): 302 (14), 288 (26), 122 (60), 115 (10), 101 (100). MS (EI, m/z, %): 146 (84), 83 (100), 55 (40). ^1^H-NMR (500 MHz, CDCl_3_, TMS): δ 2.03 (d, *J* = 1.2 Hz, 3 H, CH_3_), 2.22 (d, *J* = 1.2 Hz, 3 H, CH_3_), 5.98 (t, *J* = 1.2 Hz, 1 H, =CH), 6.73 (dd, *J* = 1.4 Hz, *J* = 8.0 Hz, 1 H, 4-H), 7.05 (dd, *J* = 1.4 Hz, *J* = 8.6 Hz, 1 H, 6-H), 7.52 (dd, *J* = 8.0 Hz, *J* = 8.6 Hz, 1 H, 5-H), 10.35 (s, 1 H, OH). ^13^C-NMR (125 MHz, CDCl_3_, TMS): 20.7 (CH_3_), 27.8 (CH_3_), 114.2 (-CH=), 116.1 (aromat. CH), 117.1 (aromat. CH), 121.4 (quat. C), 135.7 (aromat. CH), 145.8 (quat. C), 155.7 (quat. C), 162.4 (quat. C), 163.7 (CO).

### 3-Hydroxy-2-nitrophenyl benzoate (7h)

The compound was prepared according to “General Procedure 1” from 0.260 g (1.86 mmol) benzoyl chloride and 0.320 g (2.1 mmol) 2-nitroresorcinol to give 0.273 g (57%) of **7h** as a yellow solid. IR (KBr), v, cm^-1^: 3230, 2929, 2358, 1982, 1739, 1614, 1579, 1535, 1265, 1228, 1054, 702. HR-MS: Calcd. for C_13_H_9_NO_5_: 259.0481. Found: 259.0480. MS (CI): m/z (%) = 123 (88), 122 (90), 105 (100). MS (EI, m/z, %): 122 (30), 105 (100), 93 (10), 77 (60), 51 (27). ^1^H-NMR (400 MHz, CDCl_3_, TMS): δ 6.86 (dd, *J* = 1,4 Hz, *J* = 8.0 Hz, 1 H, 6-H), 7.13 (dd, *J* = 1.4 Hz, *J* = 8.6 Hz, 1 H, 4-H) 7.55 (m, 3 H, 3’-H, 4’-H, 5’-H), 7.68 (dd, *J* = 8.0 Hz, *J* = 8,6 Hz, 1 H, 5-H), 8.12 (dd, *J* = 1.4 Hz, *J* = 8.0 Hz, 2 H, 2’-H, 6’-H), 10.43 (s, 1 H, OH). ^13^C-NMR (125 MHz, CDCl_3_, TMS): 116.1 (aromat. CH), 117.6 (aromat. CH), 128.5 (quat. C), 128.6 (quat. C), 128.7 (2 aromat. CH), 130.5 (2 aromat. CH), 134.2 (aromat. CH), 135.8 (aromat. CH), 145.9 (quat. C), 156.0 (quat. C), 164.5 (CO).

### N-(2, 6-dihydroxyphenyl)-3-phenylpropanamide (8a)

The compound was prepared according to “General Procedure 2” from 120 mg (0.42 mmol) **7a** to give 78 mg (72%) of **8a** as a dark brown oil. IR (KBr), v, cm^-1^: 3288, 2925, 1707, 1637, 1601, 1533, 1341, 1266, 1036, 778. HR-MS (M^+^-1): Calcd. for C_15_H_14_NO_3_: 256.0974 ^g^/_mol_. Found: 256.0977 ^g^/_mol_. MS (EI, m/z, %): 257 (M^+^, 7), 125 (100). ^1^ H-NMR (400 MHz, d-DMSO, TMS): δ 2.80 (t, *J* = 8.2 Hz, 2 H, CH_2_), 2.97 (t, *J* = 8.2 Hz, 2 H, CH_2_), 6.39 (d, *J* = 8.2 Hz, 2 H, 2 aromat. CH), 6.87 (t, *J* = 8.2 Hz, aromat. CH), 7.14–7.22 (m, 5 H, 5 aromat. CH), 9.37 (s, 2 H, 2 OH), 9.40 (s, 1 H, NH). ^13^C-NMR (125 MHz, d-DMSO, TMS): 31.1 (CH_2_), 36.8 (CH_2_), 107.7 (2 aromat. CH). 114.2 (quat. C), 125.7 (aromat. CH), 126.1 (aromat. CH), 128.0 (2 aromat. CH), 128.0 (2 aromat. CH), 140.7 (quat. C), 151.0 (2 quat. C), 172.5 (CO).

### 6-Bromo-N-(2, 6-dihydroxyphenyl)hexanamide (8b)

The compound was prepared according to “General Procedure 2” from 0.203 g (0.61 mmol) **7b** to give 0.159 g (86%) of **8b**. IR (KBr), v, cm^-1^: 3289, 2936, 2864, 1709, 1640, 1600, 1537, 1532, 1475, 1343, 1267, 1036, 779, 719, 637. HR-MS: Calcd. for C_12_H_16_BrNO_3_: 301.0314 ^g^/_mol_. Found: 301.0311 g/mol. MS (CI, m/z, %): 304 (84), 302 (M^+^+1, 92), 224 (94), 222 (70),134 (95), 126 (100), 125 (75), 115 (43) MS (EI, m/z, %): 303 (2), 301 ([M]^+^, 2), 125 (100), 69 (17). ^1^ H-NMR (400 MHz, d-DMSO, TMS): δ 1.55 (m, 2 H, CH_2_), 1.75 (m, 2 H, CH_2_), 1.92 (m, 2 H, CH_2_), 2.55 (m, 2 H, CH_2_), 3.46 (m, 2 H, CH_2_), 6.44 (m, 2 H, 3-H, 5-H), 6.88 (m, 1 H, 4-H), 8.95 (s, 2 H, OH), 9.54 (s, 1 H, OH). ^13^C-NMR (125 MHz, d-DMSO, TMS): 23.7 (CH_2_), 26.2 (CH_2_), 31.1 (CH_2_), 32.7 (CH_2_), 34.6 (CH_2_), 107.3 (CH), 113.7 (quat. C), 125.2 (CH), 149.0 (quat. C), 172.4 (quat. C).

### 2-Cyclohexyl-N-(2, 6-dihydroxyphenyl)acetamide (8c)

The compound was prepared according to “General Procedure 2” from 232 mg (0.83 mmol) **7c** to give 184 mg (89%) of **8c** as a pale yellow solid. IR (KBr), v, cm^-1^: 3251, 2927, 1609, 1542, 1473, 1339, 1279, 1261, 1038, 778. HR-MS: Calcd. for C_14_H_19_NO_3_: 249.136494 ^g^/_mol_. Found: 249.1369 ^g^/_mol_. MS (CI, m/z, %): 250 (M^+^+1, 100), 125 (58). ^1^H-NMR (400 MHz, d-DMSO, TMS): δ 0.94–1.81 (m, 11 H, CH, 5 CH_2_), 2.31 (d, *J* = 6.8 Hz, 2 H, CH_2_), 6.35 (d, *J* = 8.3 Hz, 2 H, 2 aromat. CH), 6.87 (t, *J* = 8.3 Hz, 1 H, aromat. CH), 9.39 (s, 2 H, 2 OH). ^13^C-NMR (125 MHz, d-DMSO, TMS): 25.7 (CH_2_), 25.9 (2 CH_2_), 35.1 (CH), 3.41 (2 CH_2_), 42.9 (CH_2_), 107.7 (2 aromat. CH), 114.3 (quat. C), 126.6 (aromat. CH), 151.7 (2 quat. C), 172.6 (CO).

### 2-(Bicyclo[2.2.1]hept-2-yl)-N-(2, 6-dihydroxyphenyl)acetamide (8d)

The compound was prepared from 0.209 g (0.7 mmol) of **7d** according to “General Procedure 2” to give 159 mg (85%) of **8d**. IR (NaCl, film), v, cm^-1^: 3181, 2949, 2869, 2361, 1721, 1633, 1597, 1531, 1475, 1455, 1345, 1267, 1194, 1038, 778, 718. HR-MS: Calcd. for C_16_H_15_NO_4_: 261.1365 ^g^/_mol_. Found: 261.1368 g/mol. MS (CI, m/z, %): 262 (M^+^+1, 100), 248, (30), 210 (28), 126 (45), 125 (42). MS (EI, m/z, %): 261 (M^+^, 8), 125 (100), 95 (15), 67 (24). ^1^H-NMR (400 MHz, d-DMSO, TMS): δ 1.11 (m, 4 H, 2 CH_2_), 1.43 (m, 4 H, 2 CH_2_), 1.88 (m, 1 H, CH), 2.04 (m, 1 H, CH), 2.19 (m, 1 H, CH), 2.28 (dd, *J* = 7.7 Hz, *J* = 13.8 Hz, 1 H, CH_2_), 2.39 (dd, *J* = 8.0 Hz, *J* = 13.8 Hz, 1 H, CH_2_), 6.35 (d, *J* = 8.1 Hz, 2 H, 3-H, 5-H), 6.87 (t, *J* = 8.1 Hz, 1 H, 4-H), 9.36 (s, 2 H, OH), 9.40 (s, 1 H, NH). ^13^C-NMR (125 MHz, d-DMSO, TMS): 36.2 (CH), 42.0 (CH_2_), 107.7 (2 aromat. CH), 114.3 (quat. C), 126.6 (aromat. CH), 151.8 (2 quat. C), 172.7 (CO).

### N-(2, 6-dihydroxyphenyl)octadecanamide (8e)

The compound was prepared according to “General Procedure 2” from 100 mg (0.24 mmol) of **7e** to give 65 mg (69%) of **8e** as a brown solid of mp 98°C. IR (KBr), v, cm^-1^: 3377, 3318, 2919, 2848, 1641, 1606, 1537, 1471, 1354, 1259, 1168, 1037, 972, 815, 772, 719, 656. HR-MS: Calcd. for C_24_H_42_NO_3_: 392.3165 ^g^/_mol_. Found: 392.3158 ^g^/_mol_. MS (CI, m/z, %): 392 (M^+^+1, 100), 331 (12), 279 (6), 126 (5). ^1^H-NMR (400 MHz, d-DMSO, TMS): δ 0.86 (t, *J* = 6.8 Hz, 3 H, CH_3_), 1.24 (m, 26 H, 13 CH_2_), 1.59 (m, 2 H, CH_2_), 2.43 (t, *J* = 7.2 Hz, 2 H, CH_2_), 6.36 (d, *J* = 8.3 Hz, 2 H, 2 aromat. CH), 6.85 (t, *J* = 8.3 Hz, aromat. CH). 9.33 (s, 1 H, NH), 9.38 (s, 2 H, 2 OH). ^13^C-NMR (125 MHz, d-DMSO, TMS): 13.4 (CH_3_), 21.7 (CH_2_), 25.0 (CH_2_), 28.4 (CH_2_), 28.54 (CH_2_), 28.57 (CH_2_), 28.59 (CH_2_), 28.6 (6 CH_2_), 28.7 (CH_2_), 30.9 (CH_2_), 40.5 (CH_2_), 107.27 (2 aromat. CH), 115.5 (quat. C), 125.9 (aromat. CH), 151.1 (2 quat. C), 173.0 (CO).

### N-(2, 6-Dihydroxyphenyl)undecanamide (8f)

The compound was prepared according to “General Procedure 2” from 253 mg (0.79 mmol) of **7f** to give 164 mg (71%) of **8f** as a dark brown solid. IR (KBr), v, cm^-1^: 2925, 2854, 1709, 1602, 1532, 1468, 1347, 1268, 1038, 778. HR-MS [M^+^-1]: Calcd. for C_17_H_26_NO_3_: 292.1913 ^g^/_mol_. Found: 292.1918 ^g^/_mol_. MS (EI, m/z, %): 293 (M^+^, 3), 125 (100), 107 4). ^1^H-NMR (400 MHz, d-DMSO, TMS): δ 0.86 (t, *J* = 7.1 Hz, 3 H, CH_3_), 1.14 – 1.61 (m, 16 H, 8 CH_2_), 2.41 (t, *J* = 7.4 Hz, 2 H, CH_2_), 6.35 (d, *J* = 8.3 Hz, 2 H, 2 aromat. CH), 6.87 (t, *J* = 8.3 Hz, 1 H, aromat. CH), 9.35 (s, 1 H, NH), 9.40 (s, 2 H, 2 OH). ^13^C-NMR (125 MHz, d-DMSO, TMS): 14.0 (CH_3_), 22.1 (CH_2_), 25.4 (CH_2_), 28.5 (CH_2_), 28.7 (CH_2_), 28.8 (CH_2_), 28.9(CH_2_), 29.0 (CH_2_), 33.6 (CH_2_), 39.6 (CH_2_), 107.6 (2 aromat. CH), 126.5 (aromat. CH), 114.2 (quat. C), 151.8 (2 quat. C), 173.4 (CO).

### N-(2, 6-Dihydroxyphenyl)-3-methylbutanamide (8g)

The compound was prepared according to “General Procedure 2” from 200 g (0.8 mmol) **7e** to give 140 mg (84%) of **8e** as a brown oil. IR (KBr), v, cm^-1^: 3265, 2959, 1640, 1597, 1533, 1474, 1343, 1270, 1038, 1023, 781. ESI-HR-MS: Calcd. for C_11_H_14_NO_3_: 208.0974 ^g^/_mol_. Found: 208.0978 ^g^/_mol_. ^1^H-NMR (400 MHz, CD_2_CI_2_, TMS): δ 1.03 (d, *J* = 6.6 Hz, 6 H, 2 CH_3_), 2.19 (m, 1 H, CH), 2.36 (d, *J* = 7.4 Hz, CH_2_), 6.47 (d, *J* = 8.0 Hz, 2 H, 2 aromat. CH), 6.91 (t, *J* = 8.0 Hz, 1 H, aromat. CH), 7.96 (s, 1 H, NH), 8.85 (s, 2 H, 2 OH). ^13^C-NMR (125 MHz, CD_2_Cl_2_, TMS): 22.4 (2 CH_3_), 26.8 (CH), 46.3 (CH_2_), 109.2 (2 aromat. CH), 115.1 (quat. C), 126.7 (aromat. CH), 149.0 (2 quat. C), 173.5 (CO).

### N-(2, 6-Dihydroxyphenyl)benzamide (8h)

The compound was prepared according to “General Procedure 2” from 0.307 g (1.19 mmol) of **7d** to give 0.249 g (91%) of **8h** as a pale brown oil. IR (KBr), v, cm^-1^: 3415, 3141, 1649, 1612, 1572, 1537, 1450, 1356, 1257, 1028, 777, 696, 677, 609. HR-MS: Calcd. for C_13_H_11_NO_3_: 229.0739 ^g^/_mol_. Found: 229.0738 ^g^/_mol_. MS (CI, m/z, %): 230 (M^+^+1, 100), 126 (10), 105 (28). MS (EI, m/z, %): 229 (M^+^, 12), 105 (100), 77 (42). ^1^H-NMR (400 MHz, d-DMSO, TMS): δ 6.47 (d, *J* = 8.1 Hz, 2 H, 3-H, 5-H), 6.93 (t, *J* = 8.1 Hz, 1 H, 4-H), 7.53 (m, 3 H, 3’-H, 4’-H, 5’-H), 8.01 (d, *J* = 7.9 Hz, 2 H, 2’-H, 6’-H), 9.25 (s, 1 H, NH), 9.67 (s, 2 H, OH). ^13^C-NMR (125 MHz, d-DMSO, TMS): 107.0 (2 CH, 3-C, 5-C), 113.2 (quat. C, 1-C), 125.5 (CH, 4-C), 126.4 (2 CH, 2’-C, 6’-C), 127.3 (2 CH, 3’-C, 5’-C), 130.8 (CH, 4’-C), 131.9 (quat. C, 1’-C), 149.6 (2 quat. C, 2-C, 6-C), 165.3 (CO).
